# Accessibility of Early Infant Diagnostic Services by Under-5 Years and HIV Exposed Children in Muheza District, North-East Tanzania

**DOI:** 10.3389/fpubh.2018.00139

**Published:** 2018-05-15

**Authors:** Veneranda M. Bwana, Sayoki Godfrey Mfinanga, Edgar Simulundu, Leonard E. G. Mboera, Charles Michelo

**Affiliations:** ^1^School of Public Health, University of Zambia, Lusaka, Zambia; ^2^Amani Research Centre, National Institute for Medical Research, Muheza, Tanzania; ^3^Muhimbili Research Centre, National Institute for Medical Research, Dar es Salaam, Tanzania; ^4^Department of Disease Control, School of Veterinary Medicine, University of Zambia, Lusaka, Zambia; ^5^National Institute for Medical Research, Headquarters, Dar es Salaam, Tanzania; ^6^Strategic Centre for Health Systems Metrics and Evaluations, School of Public Health, University of Zambia, Lusaka, Zambia

**Keywords:** accessibility, children, early infant diagnosis, HIV, services, Tanzania

## Abstract

**Introduction:** Early infant diagnosis (EID) of Human Immunodeficiency Virus (HIV) provides an opportunity for follow up of HIV exposed children for early detection of infection and timely access to antiretroviral treatment. We assessed predictors for accessing HIV diagnostic services among under-five children exposed to HIV infection in Muheza district, Tanzania.

**Methods:** A cross sectional facility-based study among mother/guardian-child pairs of HIV exposed children was conducted from June 2015 to June 2016. Using a structured questionnaire, we collected information on HIV status, socio-demographic characteristics and other relevant data. Multiple regression analyses were used to investigate associations of potential predictors of accessing EID services.

**Results:** A total of 576 children with their respective mothers/guardians were recruited. Of the 576 mothers/guardians, 549 (95.3%) were the biological mothers with a median age of 34 years (inter-quartile range: 30–38 years). The median age of the 576 children was 15 months (inter- quartile range: 8.5–38.0 months). A total of 251 (43.6%) children were born to mothers with unknown HIV status at conception. Only 329 (57.1%) children accessed EID between 4 and 6 weeks of age. Children born to mothers with unknown HIV status at conception (AOR = 0.6, 95% CI 0.4–0.8) and those with ages 13–59 months (AOR = 0.4, 95% CI 0.2–0.6) were the significant predictors of missed opportunity to access EID. Children living with the head of household with at least a high education level had higher chances of accessing EID (AOR = 1.8, 95% CI 1.1–3.3). Their chances of accessing EID services was three-fold higher among mothers/guardians with good knowledge of HIV infection prevention of mother to child transmission (AOR = 3.2, 95% CI 2.0–5.2) than those with poor knowledge. Mothers/guardians living in rural areas had poorer knowledge of HIV infection prevention of mother to child transmission (AOR = 0.6, 95% CI 0.4–0.9) than those living in urban areas.

**Conclusion:** Accessibility of EID services among children below 5 years exposed to HIV infection in Muheza is low. These findings stress the need for continued HIV education and outreach services, particularly in rural areas in order to improve maternal and child health.

## Introduction

Worldwide, human immunodeficiency virus (HIV) is considered as one of the most important infectious pathogen afflicting children. In 2016 it was estimated that 2.1 million children were living with HIV and more than 90% were in sub-Saharan Africa (SSA) (1). In the same year, approximately 160,000 new HIV infections, which occurred in children worldwide, were due to mother to child transmission of HIV (MTCT), either during pregnancy, childbirth or breast feeding (1). Most infected infants and children die from HIV-related causes without being tested of the infection. Without access to cotrimoxazole prophylaxis, antiretroviral therapy (ART) and supportive care, about 40% of HIV-infected infants in developing countries progress to death within 1 year of age and 50% within 2 years of age (2, 3). Studies have demonstrated a remarkable increase in survival rate if HIV-infected children have access to early HIV diagnosis and treatment (4).

Early Infant Diagnosis (EID) of HIV infection is part of maternal and child health service package that has been integrated into Prevention of Mother to Child Transmission of HIV (PMTCT) interventions since 2006 in most countries in SSA including Tanzania (5–9). EID using deoxyribonucleic acid-polymerase chain reaction (DNA-PCR) testing for HIV infection provides a definitive diagnosis in children less than 18 months of age (10, 11). The World Health Organization (WHO) recommends early testing for HIV DNA between 4 and 6 weeks of age to all infants born to HIV positive mothers (8, 12). EID provides an opportunity to identify HIV infected children for early clinical evaluation, prophylaxis for opportunistic infections and antiretroviral therapy (ART) aiming to reduce morbidity and mortality (4). However, late diagnosis of pediatric HIV infections and persistence of vertical transmission continues to be the major challenge despite the introduction of PMTCT services. In 2014, WHO estimated that only 50% of all HIV-exposed infants accessed EID services within 2 months of age (3). This is still far below the 80% EID services coverage recommended by WHO (8). These statistics suggest that accessibility to EID programs is still a challenge in resource-limited settings (13). Majority of HIV-exposed and HIV-infected children remain unidentified due to inaccessibility and insufficient utilization of these services (14).

According to a WHO report of 2016, MTCT accounts for about 18% of the new HIV infections in Tanzania (15). Good progress has been made in scaling up the quality of PMTCT services in Tanzania. More than 90% of Reproductive and Child Health (RCH) clinics are providing PMTCT services (12). The Tanzania National AIDS Control Program (NACP) started HIV care and treatment services in 2004. It was estimated that more than 300,000 children below 15 years old were living with HIV in 2013 and only 16% of children estimated to be eligible for ART were receiving treatment by 2013 (16). The major contributing factors to the low ART coverage in infants and children in the country might be due to limited capacity of most health facilities to conduct virological testing (17). Nevertheless, the goal of EID is to identify early HIV infection prior to progression of clinical disease and referral of all HIV infected infants to HIV Care and Treatment Centres (CTC) (12). This is being done by collecting dried blood spots (DBS), which are then sent to four zonal laboratories located within referral hospitals, namely Kilimanjaro Christian Medical Centre (KCMC), Muhimbili National Hospital, Mbeya Referral Hospital and Bugando Medical Centre to be tested for HIV infection using DNA-PCR testing as part of EID services (18). By December 2016, there were 4,737 sites providing EID services in Tanzania scaled up to involve urban and rural areas in all regions (19).

Studies in Mozambique and Tanzania have shown that only 25 and 67% of infants who were registered for care were brought for EID, respectively (20, 21). In Mozambique about a quarter of HIV exposed infants received the first HIV test at a median age of 5.0 months (21) while in Zambia nearly three quarters received the test at a median age of 8.1 months (22). Accordingly, the median age at first HIV test was 5.0 months in Uganda (23) while in Cameroon it ranged from 1.5 to 4.0 months (24, 25). In contrast, in Nigeria, the median age was 13 weeks (range = 4.0–72 weeks) (6) while studies in Tanzania reported the median age of 5.6–16.0 weeks at first HIV test (9, 20, 26).

Overall, these studies suggest the existence of some variability at the age of first HIV test among children within the African region (27). In previous studies, stock out of supplies, limited laboratories that can perform PCR analysis, weak infrastructures and inadequate health personnel trained in DBS techniques were identified as challenges for effective EID services implementation in most countries in SSA (28–30). Maternal awareness of HIV control and prevention, cost of transport, poverty, stigma and discrimination (5, 7, 28, 31) were reported to affect access to EID services. Therefore, the main gaps concerning EID services are low coverage and timely testing at first HIV test as compared to 80% coverage and testing at the age of 4–6 weeks, respectively, as recommended by WHO. Consequently, EID services are still challenging in resource-limited countries in SSA including Tanzania, where only a few HIV exposed infants are tested (3, 21, 32). This study aimed to assess predictors of mothers/guardians to obtain EID services for children aged under 5 years exposed to HIV infection in Muheza district, Tanzania. In the context of this study, a guardian was defined as the child's main primary caregiver living with the child in the same household and this included either the biological parent, grandparent, sister, brother, aunt or uncle.

## Materials and methods

### Study area

The study was conducted in Muheza district north-eastern Tanzania (4°, 45′S; 39°00′E). The district has a total of 46 health facilities (one district hospital, 4 health centers and 41 dispensaries); 42 of which provide RCH services, 36 offer PMTCT services and 28 offer EID services (33). The prevalence of HIV in the district as reported in 2013 was 3.9% (34). The HIV prevalence among pregnant women in Muheza district in 2015 was 4.0% higher than that of its neighboring districts as detailed in the Supplementary Material (35). Muheza district was selected for this study because it is among the leading districts in Tanga Region with high HIV prevalence among pregnant women (35). The study was conducted in 18 health facilities (one district hospital, three health centers and 14 dispensaries).

### Study design, population and sampling

This was a cross-sectional study using a multi-stage sampling approach conducted during the period June 2015 to June 2016. In the first step, we aimed to select a total of 18 out of 46 health facilities in the district as detailed in the Supplementary Material. The health facilities defined the primary sampling units (PSUs)/clusters. A list of 46 PSUs was obtained from Muheza District Council and the facilities were numbered from one to 46 according to their geographical location. Then the sampling interval was obtained and 18 health facilities were randomly selected from the list. During the selection process, health facilities that were selected from the list and were found to have no EID services were removed and the next on the list was included. The process was repeated until all 18 facilities that were required for the study were included. The study population included all selected mothers/guardians with children below 5 years born to HIV positive mothers who were not breast feeding for ≥6 weeks. The sample size was calculated based on the formula that accounted for simple random sampling and the design effect which accounted for between and within cluster variation (36). We assumed the highest exposure of infection risk and a response rate of 90% and thus the estimated sample size was 836 children.

### Data collection

Four nurse counselors were trained on how to conduct interviews using a pre-tested questionnaire. Most of the questions were closed with an open option, and some were open-ended. Information on HIV status, socio-demographic characteristics and other relevant family and health status were collected. Socio-demographic information included age, sex, religion, residence, income, education, marital status, occupation, and size of household. Further information collected included knowledge on HIV transmission and prevention, time period when the child's first HIV test ought to be performed, distance to the nearest health facility (which was defined by time taken in minutes to reach the facility on foot) and maternal information which included whether the index pregnancy was planned or not, as well as HIV status prior to conception.

Mothers/guardians' satisfaction on services at health facility was assessed based on five key questions. The questions were mainly addressing the mother/guardian's perceptions regarding health care services such as HIV testing, treatment, advice, nutritional and social support received at the health facility. Each question had five options of pre-coded responses with a neutral point being neither satisfied nor dissatisfied, or neither poor nor good. Moreover, the general quality of all health services was assessed on how low or high they were satisfied with the overall quality of health care services provided at a particular health facility. This included services as a whole, reception by health workers, comfortability with the environment of care, freedom to interact with health personnel (asking questions for clarification), family planning, adequate medicines and diagnostic services. Mother/guardian's willingness to receive the HIV testing and diagnosis for their children was also assessed.

Mother/guardian's knowledge and beliefs on HIV prevention and transmission including MTCT of HIV was also assessed based on four key questions. These questions were mainly addressing general HIV transmission, timing of post exposure prophylaxis to HIV exposed infant, factors affecting HIV transmission and prevention on the risk of MTCT. Mothers/guardians whose children received an HIV test late (i.e., testing at ≥7 weeks of age) were asked to state the reasons for the delay. Multiple responses regarding the factors associated with missed opportunity to receive the first HIV test for their children at the age between 4 and 6 weeks were assessed. During recruitment, more data of the child and her mother were extracted from registers, child card and hospital case files to supplement the collected primary data.

### Laboratory tests

The HIV DNA PCR testing was used to confirm HIV infection in all HIV exposed children less than18 months of age according to national guidelines. DBS samples were routinely collected from HIV exposed children at the first encounter to the health facility. For infants, using a heel prick, five circles were filled with blood on a specific Whatman filter paper card and air dried for ≥4 h on a drying rack. After drying, the cards were placed in a gas-impermeable zip locked bag with desiccant sachets and stored in a safe location. All DBS samples from peripheral health centers and dispensaries were transported to the district hospital and then were mailed to the KCMC Clinical Laboratory in Moshi, Tanzania. In the laboratory, the DBS were tested for HIV DNA using version two of the COBAS AmpliPrep/COBAS TaqMan 48 system. One DBS circle was used to run a DNA-PCR test and if positive, a second circle was analyzed to confirm the first result. A test result was considered positive if both PCR assays were positive. The Rapid HIV antibody test was done in all HIV exposed children aged more than 18 months using Determine®/Bioline® rapid tests for the first test. If the first test was positive, the second test was performed using Uni-Gold® rapid test. HIV diagnosis was confirmed based on concordance of the results of these two HIV rapid tests. Furthermore, the sample turnaround time (TAT) which was defined as time between DBS sample collection and return of HIV DNA PCR results to the facility, for the first HIV DNA PCR test was assessed.

### Data management and analysis

To facilitate data entry, responses provided in open-ended questions were re-coded into themes which were developed to respond to study objectives. Data were double entered in Epi Data database version 3.1 (http://www.epidata.dk/) and then transferred to STATA version 13 statistical package (Stata Corp, College Station, Texas, USA). Continuous variables were described using median and inter quartile range (IQR). Categorical variables were described using frequencies and percentages. The accessibility to first HIV test by the child was categorized into a binary variable (dependent variable), “yes” as obtained or “no” as not obtained the test at the age of 4–6 weeks. A composite variable on knowledge and beliefs on HIV prevention and MTCT of the mother/guardian was generated based on the four key questions as described above. The knowledge level was categorized into a binary variable “good” or “poor.” Guardians' perceptions to healthcare services based on five options of pre-coded responses with a neutral point being neither satisfied nor dissatisfied, or neither poor nor good were described by using percentages.

Binary logistic regression was done and all factors with *p*-values of ≤ 0.2 including priori factors were considered for multiple variable logistic regression analysis. Multiple logistic regression analyses were used to examine the associations between various factors (including child and maternal, guardian, head of household characteristics) and service accessibility by the child at the age of 4–6 weeks. A manual backward stepwise selection was employed by removing non-significant variables (one at a time starting with those with the highest *p*-value). Goodness of fit of the final model was tested using likelihood ratio test. The final model consisted of variables that were significant at *p*-value of ≤ 0.05 including those with epidemiological importance. Adjusted odds ratios with their corresponding 95% confidence intervals were estimated and presented. Three individual multiple regression models were fitted for (1) the child and maternal variables, (2) guardian variables, and (3) the head of household variables. Subsequently, two combined models were fitted: (1) a model that contains the three set variables; and (2), a model that includes only significant variables which was used in the final interpretation. In addition, a separate model to assess the mother/guardian's knowledge of HIV (including MTCT and PMTCT) was also fitted. In this study, “accessibility of first HIV test by the child at the age of 4–6 weeks” was defined as the time when the child was obtaining HIV test at the age of 4–6 weeks.

### Ethical considerations

Ethical approval was obtained from the Medical Research Coordinating Committee of the National Institute for Medical Research in Tanzania (Ref: NIMR/HQ/R.8a/Vol. IX/1978) and University of Zambia Biomedical Research Ethics Committee (Ref: 001-01-15). Permission to conduct this study was given by Muheza District Council Authority. Written consent was obtained from each guardian before recruitment. Counseling of the mothers/guardians of HIV-exposed children before and after the HIV test was performed in accordance with standard testing and counseling guidelines. In this study the HIV status for all HIV exposed children was determined for effective appropriate care and management. All confirmed HIV positive children were referred to CTC for initiation of ART in accordance with Tanzania guidelines on the management of HIV and AIDS.

## Results

### Baseline characteristics

A total of 576 mother/guardian-child pairs were included in the study. The median age of the mothers/guardians included in the study was 34 years (IQR: 30–38 years). Majority (95.3%) were the biological mothers. Thirteen (2.3%) children were living with grandmothers as their guardians. Most (70.1%) of the head of household were reported to be males. Of the children included, 51.2% were females. The median age was 15 months (IQR: 8.5–38). A total of 329 (57.1%) children received the first HIV test between 4 and 6 weeks of age. The overall median age at the time of first HIV test was 6 weeks (IQR: 6–20 weeks). Sixty-one (10.6%) children were found to be HIV- positive of which 31were confirmed by HIV DNA test, and 30 by HIV antibody test. Majority (67.2%, *n* = 41) of HIV positive children were diagnosed at ≥13 months of age. Of the 61 HIV positive children, the overall median age at diagnosis was 20 months (IQR: 12.5–35 months). Accordingly, four (6.5%), 15 (24.6%), and 42 (68.9%) HIV positive children were ≤ 12 months, 13–24 months and 25–59 months age group, respectively. Out of 576 children, 43.6%, *n* = 251) were born to mothers with unknown HIV status at conception. A proportion of women (5.9%, *n* = 34) in this study never had an HIV test before and even during their index pregnancy. Some (1.9%, *n* = 11) mothers were tested after their sick children tested positive when seeking care at a health facility. Nevertheless, a number of these mothers did not know their HIV status before being pregnant and were labeled as “unknown HIV status at conception” (Table 1).

**Table 1:** Demographic distribution of the study participants.

**Children**	***n* (%)**
Age category(months)	
≤12	224 (38.9)
13–24	155 (26.9)
25–59	197 (98.8)
Sex	
Male	281 (48.8)
Female	295 (51.2)
Residence	
Rural	445 (77.3)
Urban	131(22.7)
**Guardians**	
Age category(years)	
15–24	58 (10.1)
25–70	518 (89.9)
Sex	
Male	7 (1.2)
Female	569 (98.8)
Education	
No education	78 (13.5)
Primary complete	451 (78.3)
Secondary/high school and above	47 (8.2)
Marital status	
Married/living together	519 (90.1)
Separated/divorced/widow	57 (9.9)
Occupation	
Trading	97 (16.8)
Formal employment	15 (2.6)
Subsistence farmer	464 (80.6)
Income	
Low (≤ 34 US Dollar)	45 (7.8)
High (>34 US Dollar)	531 (92.2)
Relation to the child	
Mother	549 (95.3)
Father	7 (1.2)
Grandmother	13 (2.3)
Aunt	7 (1.2)
**Heads Of Household**	
Age category (years)	
15–24	31 (5.4)
25–85	545 (94.6)
Sex	
Male	404 (70.1)
Female	172 (29.9)
Education	
No education	58 (10.0)
Primary complete	46 (8.0)
Secondary/high school and above	474 (82.0)
Relation to the child
Mother	65 (11.3)
Father	355 (61.6)
Grandmother/grandfather	131 (22.7)
Other relatives^‡^	25 (4.4)

(a) Total sample size, N = 576. (b) Guardian in this study was the main primary caregiver of the child living in the same household. (c)

‡ *Other relatives included were the sister, brother, aunt and uncle*.

### Turnaround time

Out of 576 children, 88.5% (*n* = 510) children received the first HIV DNA PCR test. Only 413 records had complete data on the date of sample collection and the date of arrival of the DNA PCR results to the health facility. The date of arrival of PCR results to health facility was not recorded in 97 records. The sample TAT for first HIV DNA test for the 413 records examined was 6 weeks (IQR: 5–10 weeks). In addition, some mothers/guardians were not satisfied with the receipts of HIV results for their children due to variability in TAT. Some (12.5%, *n* = 72) were discouraged to return to a health facility to collect their children's results. Three (0.5%) guardians each with HIV exposed child aged 6 months, 9 months, and 4 years, respectively declared that they have never received the first DBS results of their children since birth. Others (1.0%, *n* = 6) claimed that their results were not seen or found at the clinic.

### Predictors associated with accessibility of EID services

In multiple logistic regression models for individual characteristics, we found that children with mothers/guardians who were married/living together with their spouses, having general good knowledge on HIV prevention and transmission from mother to child were statistically significant predictors of accessing EID at the age of 4–6 weeks. Additionally, children with the head of household as the father who had attained secondary or high school education were significantly associated with timely access to EID services (Table 2). Mothers/guardians with good knowledge on HIV, had significantly higher odds ratio for the mother/guardian-child pairs accessing EID (AOR = 3.0, 95% CI 1.7–4.8) compared to those with poor knowledge. For every mother/guardian who was married/living together with their spouses, the odds ratio of mother/guardian-child pairs accessing EID increased (AOR = 2.3, 95% CI 1.2–4.6) compared to those who were separated/divorced. Those with the head of household as the father (AOR = 2.1, 95% CI 1.2–3.6) and who has attained secondary or high school education (AOR = 1.8, 95% CI 1.2–3.3), had significantly higher odds for mother/guardian-child pairs accessing EID (Table 2).

Table 2Multiple logistic regression of predictors of HIV testing of children at the age of 4–6 weeks in Muheza by.

**Table:** 

**Variables**	**AOR(95% CI)**
**(i) Child and maternal characteristics**	
Age category(months)	
≤12	1
13–24	0.4 (0.2–0.6)^*^
25–59	0.3 (0.2–0.5)^*^
Sex	
Female	1
Male	1.0 (0.7–1.5)^**^
Residence	
Urban	1
Rural	1.1 (0.7–1.8)^**^
Place of delivery	
Home	1
Health facility	0.6 (0.3–1.2)^**^
HIV status	
Negative	1
Positive	0.2 (0.1–0.4)^*^
Maternal HIV status at conception	
Known	1
Unknown	0.6 (0.4–0.8)^*^
Maternal planned index pregnancy	
Yes	1
No	0.7 (0.5–0.9)^*^
Unknown	0.4 (0.1–1.3)^**^

(a)

* *Significant variables based on P-value*,

** *Not significant at P-value ≤ 0.05*.

*(b) AOR, Adjusted odd ratio; CI, Confidence interval*.

**Table:** 

**Variables**	**AOR(95% CI)**
**(ii) Guardian characteristics**	
Age category(years)	
25–70	1
15–24	1.1 (0.6–2.3)^**^
Education	
No education	1
Primary complete	0.7 (0.4–1.3)^**^
Secondary/high school and above	0.9 (0.4–2.5)^**^
Marital status	
Separated/divorced/widow	1
Married/living together	2.3 (1.2–4.6)^*^
Reported age to perform first test	
4–6 weeks	1
≤1 week, 2–240 weeks	0.07 (0.04–0.13)^*^^§^
I don't know	0.2 (0.1–0.3)^*^
Knowledge on HIV	
Poor	1
Good	3.0 (1.7–4.8)^*^
Monthly income	
High (>34 US Dollar)	1
Low (≤34 US Dollar)	1.6 (0.7–3.7)^**^
Size of the household	
≤7 people	1
>7 people	0.5 (0.2–0.9)^*^
Distance to health facility	
Near (≤30 min)	1
Far (>30 min)	0.5 (0.3–0.7)^*^

(a)

* *Significant variables at P-value ≤ 0.05*,

** *Not significant at P-value ≤ 0.05*.

(b) AOR, Adjusted odd ratio; CI, Confidence interval. (c)

§ Unable to round off.

**Table:** 

**Variables**	**AOR(95% CI)**
**(iii) Head of household characteristics**	
Age category (years)	
25–85	1
15–24	1.1(0.5–2.4)^**^
Education	
No education	1
Primary complete	1.6 (0.7–3.6)^**^
Secondary/high school and above	1.8 (1.2–3.3)^*^
Relation to the child	
Mother	1
Father	2.1(1.2–3.6)^*^
Grandmother/grandfather	1.0 (0.5–1.8)^**^
Other relatives^‡^	1.5 (0.6–3.7)^**^

(a)

* *Significant variables at P-value ≤ 0.05*,

** *Not significant at P-value ≤ 0.05*.

(b) AOR, Adjusted odd ratio; CI, Confidence interval. (c)

‡ *Other relatives included were the sister, brother, aunt and uncle*.

In contrast, having a child in the age groups of 13–24 months (AOR = 0.4, 95% CI 0.2–0.6) or 25–59 months (AOR = 0.3, 95%CI 0.2–0.5), and a child being found HIV positive (AOR = 0.2, 95% CI 0.1–0.4) were associated with lower odds of timely access to EID services (i.e., they did not obtain HIV test at 4–6 weeks of age). Similarly lower odds to access EID were observed among children born to mothers with unplanned pregnancies (AOR = 0.7, 95% CI 0.5–0.9) and those born to mothers with unknown HIV status at conception (AOR = 0.6, 95% CI 0.4–0.8). Likewise, lower odds to access EID were also observed among children with mothers/guardians who did not know the age when the first HIV test of the child ought to be performed (AOR = 0.2, 95% CI 0.1–0.3); and those who lived in areas located far away from a health facility (AOR = 0.5, 95% CI 0.3–0.7) (Table 2).

However, in a separate multiple variable analysis that combined all variables, we found that children with mothers/guardians who were married/living together with their spouses (AOR = 2.3, 95% CI 1.2–4.6), having general good knowledge on HIV (AOR = 2.4, 95% CI 1.4–4.0) remained independently associated with higher chances of accessing EID services. Having a child in the age groups of 13–59 months (AOR = 0.4, 95% CI 0.2–0.7), a child being found HIV positive (AOR = 0.3, 95% CI 0.1–0.6), living far away from the health facility (AOR = 0.6, 95% CI 0.4–0.9) and mothers/guardians who did not know the age when the first HIV test of the child ought to be performed (AOR = 0.2, 95% CI 0.1–0.4) remained independently associated with lower chances to access EID services (Table 3).

**Table 3:** Multiple logistic regression results of predictors of HIV testing of children at the age of 4–6 weeks in Muheza district, Tanzania.

**Children**	**AOR(95%CI)**
Age category(months)	
≤ 12	1
13–24	0.4 (0.2–0.7)^*^
25–59	0.4 (0.2–0.7)^*^
HIV status	
Negative	1
Positive	0.3 (0.1–0.6)^*^
**Guardians**	
Marital status	
Separated/divorced/widow	1
Married/living together	2.3 (1.2–4.6)^*^
Reported age to perform first test	
4–6 weeks	1
≤ 1 week, 2–240 weeks	0.08 (0.04–0.14)^*^^§^
I don't know	0.2 (0.1–0.4)^*^
Knowledge on HIV	
Poor	1
Good	2.4 (1.4–4.0)^*^
Distance to health facility	
Near (≤30 min)	1
Far (>30 min)	0.6 (0.4–0.9)^*^

(a)

* Significant variables at P-value ≤ 0.05. (b) AOR, Adjusted odd ratio; CI, Confidence interval. (c)

§ *Unable to round off*.

### Knowledge of HIV transmission and prevention

Results of the multiple logistic regression model (Table 4) showed significant association between general knowledge on HIV (including MTCT and PMTCT) and level of education, residence and marital status. The mothers/guardians who were married or living together with their spouses (AOR = 2.8, 95% CI 1.6–4.9) and with a primary education (AOR = 1.8, 95% CI 1.1–3.1) had good knowledge on HIV (including MTCT and PMTCT). Mothers/guardians living in rural areas had poor knowledge on HIV (including MTCT and PMTCT) (AOR = 0.6, 95% CI 0.4–0.9).

### Associated barriers to access EID services

A total of 247 mothers/guardians whose children missed the first HIV test between 4 and 6 weeks of age were also interviewed. Factors that hindered access to EID services included inadequate knowledge and awareness of EID services (25.5%), children appeared to be in good health (13.8%) and so the guardians felt there was no need to access the services, lack of paternal permission or support (12.6%) and some mothers feared the possibility of their children being found HIV positive (5.4%). Stock out of DBS kits (6.5%), unavailability of trained staff (2.0%), poor DBS sample collection techniques (2.8%) and lack of knowledge among health workers on the correct age when the first HIV test of the child is supposed to be performed (4.5%) were found to affect access to EID services. In addition, three mothers refused to take ARV and said that they believed in the Almighty God who will cure them from HIV infection through prayers. Seventeen mothers did not inform their spouses about their HIV status. These attitudes and beliefs deterred women from seeking PMTCT services which led to poor adherence to PMTCT interventions including accessing EID services (Table 5).

**Table 4:** Guardian characteristics associated with general good knowledge on HIV transmission and prevention from mother to child.

**Guardians**	**AOR(95%CI)**
Age category(years)	
25–70	1
15–24	2.0 (0.9–4.4)^**^
Sex	
Female	1
Male	0.2 (0.1–1.2)^**^
Residence	
Urban	1
Rural	0.6 (0.4–0.9)^*^
Education	
No education	1
Primary complete	1.8 (1.1–3.1)^*^
Secondary or high school and above	0.7 (0.3–1.5)^**^
Marital status	
Separated/divorced/widow	1
Married/living together	2.8 (1.6–4.9)^*^

(a)

* *Significant variables at P-value ≤ 0.05*,

** *Not significant at P-value ≤ 0.05. (b) AOR, Adjusted odd ratio; CI, Confidence interval*.

**Table 5:** Barriers associated with accessibility to EID services in Muheza.

**Individual factors**	***n* (%)**
Culture issues^#^	11 (4.5)
Children appeared in good health	34 (13.8)
Inadequate knowledge and awareness on EID services	63 (25.5)
Long distance to the health facility	9 (3.6)
Lack of money for transport to go to the health facility	13 (5.3)
Lack of paternal permission/support to access EID services	31 (12.6)
Guardian was uninformed if the child was born to HIV+ mother	13 (5.3)
Guardian-child pairs relocated to a residence far away from the facility with EID service	9 (3.6)
Their mothers disbelieve their HIV+ results and were not on ARV during pregnancy^†^	7 (2.8)
Mothers feared that their HIV+ status will be known by health workers^*^	14 (5.4)
Mothers feared if their children would be found to be HIV+^*^	11 (4.5)
**Health facility factors**	
Long waiting time to get services at the facility	6 (2.4)
Health workers did not know the correct age to perform child's first HIV test	11 (4.5)
Mothers not registered at the health facility, so the child was not tested	6 (2.4)
The child's clinic card did not have facility number, so he was not tested	10 (4.0)
Unavailability of trained health workers	5 (2.0)
Stock out of DBS kits	16 (6.5)
Improper collection of DBS specimen	7 (2.8)
The first HIV test results lost	4 (1.6)

(a)

# The cultural does not allow to send back the young child to the health facility, the cultural does not allow the young child to be carried by everyone and should be kept inside the house. (b)

† Three mothers believe in Almighty God who will cure them from HIV infection through prayers and refuse to take ARV. (c)

* *HIV related stigma experienced by 17 mothers has directly affect access to EID services*.

### Satisfaction of mothers/guardians on health care services

**Figure 1:**
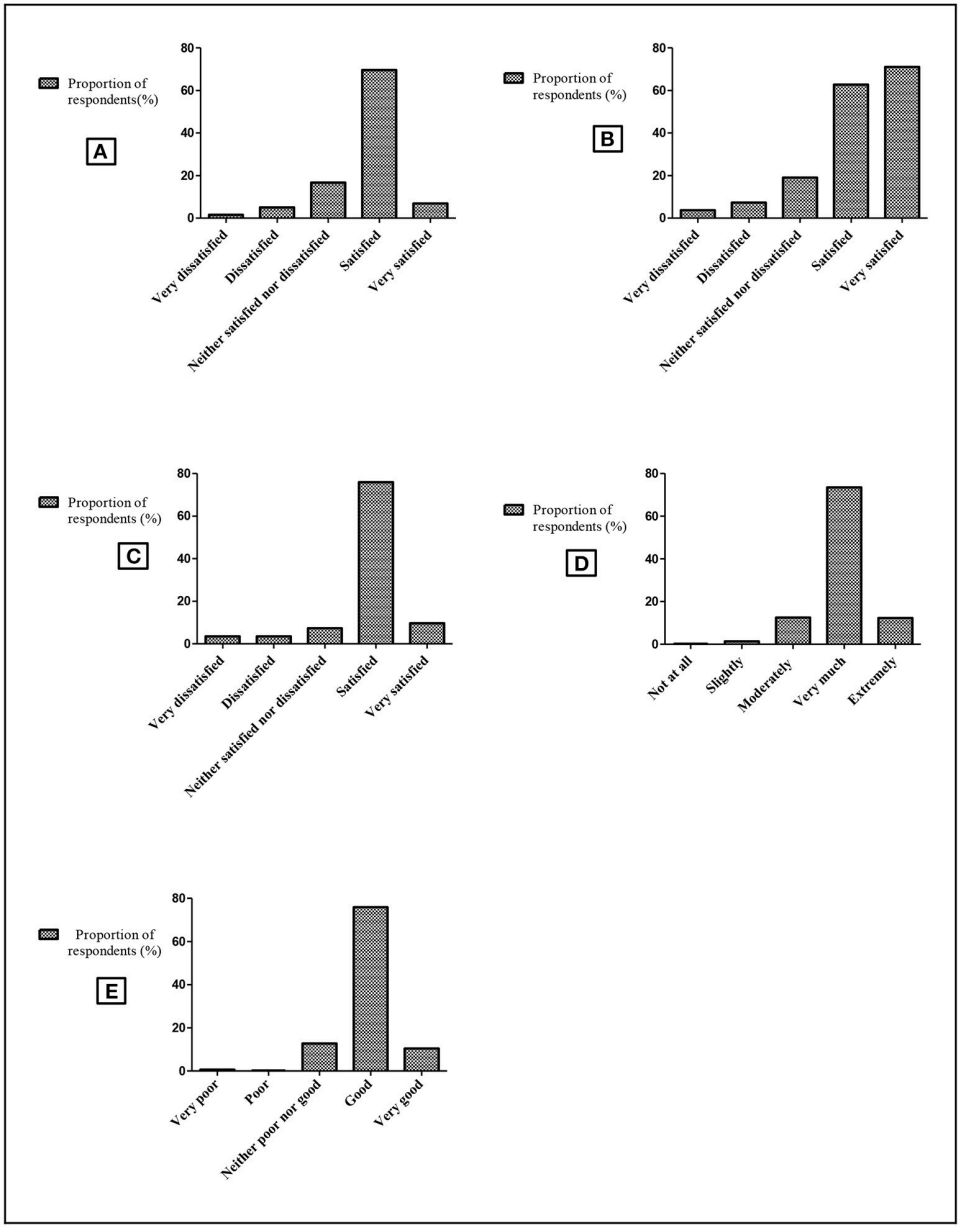
Mothers/guardians' satisfaction by health care services in Muheza. Schematic presentation showing mother/guardian's perceptions regarding receiving services at the health facility such as level of care, HIV testing, treatment and advice. The horizontal axis shows satisfaction levels as a rating scale of five options. The vertical axis shows proportion of respondents (mothers/guardians) corresponding to the 5 options on each column. **(A)** Level of satisfaction with the reception given by health care workers. **(B)** Level of satisfaction of care at the facility (space, confidentiality, attention). **(C)** Level of satisfaction with EID services provided to their children. **(D)** Trustworthiness on health care workers' information (care, nutritional, social support). **(E)** Level of overall quality of health care services at the facility.

A total of 576 guardians were asked about their satisfaction with the quality of services offered at health facilities. About 402 (69.8%) mothers/guardians were satisfied with the level of care at the facility in terms of space, confidentiality and attention. Most of them (85.5%, *n* = 492) were satisfied with EID services provided to their children (Figure 1). Generally, low satisfaction with the overall quality of services at the facility was reported by 306 (53.1%) mothers/guardians. However, 559 (97.1%) mothers/guardians were willing to receive EID services for their children.

## Discussion

We observed that only slightly over half of the children below 5 years exposed to HIV infection accessed EID services between 4 and 6 weeks of age in Muheza district. The EID services coverage in Muheza is still below the 80% threshold recommended by WHO (8). However, the coverage has increased compared to previous reported data from Tanzania and some countries in SSA which ranged from 4 to 55% (24, 27, 37–39). Nevertheless, EID services coverage is considerably lower when compared to studies conducted in Botswana, Swaziland, South Africa and Malawi which ranged from 58 to 94% (27, 38, 40, 41).

Several factors affecting access to EID including both individual and health facility (institutional) were identified. At the individual level, inadequate maternal knowledge and awareness of EID services, lower levels of education of the head of household, lack of paternal support/permission, larger size of household, cost of transport, long distance to health facilities and HIV-related stigma were identified as barriers of accessing EID services. At the health facility level, unavailability of trained staff, inadequate supplies of laboratory materials and late return of HIV test results were the main constraining factors.

Older children and those who were HIV infected had accessed the first HIV test at ≥ to 7 weeks of age. The observed late accessibility to HIV testing and late HIV diagnosis in older children was also consistent with other studies (42, 43). This implies that accessibility to HIV testing among children at older ages could have been prompted by manifestation of clinical symptoms that required diagnostic testing. This suggests that the current recommended age to access EID should be evaluated and if feasible to start performing the first HIV test within few days after delivery. The strategy will create awareness and increase motivation to bring the child later at the age of 6 weeks and EID coverage will be increased. This will ensure early enrolment into continuum of care in order to identify early HIV infection for timely access to ARV treatment.

Children born to mothers with unknown HIV status at conception were among those who missed an opportunity to access EID services in Muheza. Several studies reported that routine HIV testing to all infants presented at vaccination clinics (27, 44–48) and to all hospitalized children (49, 50) have been found to identify large number of HIV exposed infants and children. In Botswana, Uganda and Malawi, EID of HIV is offered even to those infants born to mothers of unknown HIV status (51–53). In contrast, in Mozambique, EID programs target infants born to mothers with known HIV positive status (21). These studies indicate that a considerable number of HIV infected children will remain unidentified in the community if EID services are offered only to children born to mothers with known HIV status.

In our study, higher chances to access EID services were observed among children whose heads of household had attained high education level. Studies conducted elsewhere reported that parents with low educational status tend to have poor follow up to scheduled clinic visits (28, 54, 55). In addition, inadequate mother/guardian's knowledge and awareness of HIV/AIDS observed in our study, have also been shown to affect access to EID in South Africa (56). Interestingly, a number of HIV exposed children were living with grandmothers as their main primary caregivers in Muheza. Similar finding have been reported elsewhere (56, 57). These grandmothers may be unaware of EID services or may not know the importance of regular child follow up to EID services. As such community awareness and understanding of the ongoing HIV and AIDS interventions programs should be emphasized to every individual, including grandmothers.

In our study, the long distance to the health facility, larger size of the household, affordability to cater for transport costs and paternal permission/support were found to affect access to EID. Similar factors have been reported elsewhere (21, 28, 37, 41, 49, 50, 57, 58). These findings suggest the need to develop and strengthen the health related funding mechanisms that will improve social networks and support structures in the community. In addition, HIV-related stigma, such as failure by a maternal spouse to disclose their HIV status to their family, maternal fear of the possibility of their child being found HIV positive were found to affect access to EID services in Muheza District. This results into poor compliance to PMTCT and EID services and may lead to poor attendance of regular clinic visits for their children. These findings were also similar to those reported previously (31, 55).

Health facility factors were also found to affect EID services in this study. Poor DBS sample collection techniques and lack of knowledge of the age when the first HIV test of the child ought to be performed among health workers were observed to affect EID. Stock out of laboratory materials like DBS kits was also a barrier to access EID services in the district. Similar findings have been documented in other studies (28, 30, 39, 55). Provision of continuous training especially in PMTCT and EID services to health personnel with first priority being given to those involved in providing pediatric care is highly needed. Furthermore, the long TAT of PCR results have been shown to affect access to EID services in our study. The finding is consistent with those reported from other studies (22, 23, 25, 28, 47). The long TAT definitely leads to delay in making early diagnosis for early initiation of ART for HIV infected children, whilst continuing with routine care for HIV uninfected children.

This study has limitations, including the potential for reporting bias of the date of first HIV test and what transpired during the pregnancy of their index children. Of note, reporting bias was observed in those children who had received the first HIV test before initiation of our study. But this was minimized through verification using the children's under five or antenatal cards, the antenatal care (ANC) records, PMTCT records and laboratory DBS forms. The records were compared to the responses given by the respondents. Stratified random selection could have been the best approach in this study, but this would have entailed to enroll more facilities per strata, and considering the limited resources we opted not to stratify. A total of 28 guardians with HIV exposed children below 5 years refused to participate in the study. These mothers/guardians-child pairs may have different characteristics compared to those who participated in the study. Finally, while factors included in our multiple variables logistic model were each potentially important predictors of accessibility to EID services, we cannot eliminate the probability that our results were manipulated by unmeasured confounders.

## Conclusion

Accessibility to HIV testing and diagnostic services among children exposed to HIV infection in Muheza district is low despite the investment made in the past decade. This could be partly due to poor knowledge of PMTCT among mothers/guardians. The findings underscore the need to focus on effective implementation of PMTCT programs in order to improve EID services in similar settings. More strategies are needed to evaluate the age for entry point to access EID services in order to increase EID coverage. In addition, community awareness and education of the ongoing HIV and AIDS interventions programs, training of health workers especially in PMTCT and EID services should be improved. Educational campaigns that provide massages on the risk factors of MTCT and the importance of follow up appointments to EID clinics need to be emphasized.

## Author contributions

VB conceived and conducted the study. VB, CM, LM, SM, ES contributed to the study design and analysis of results. CM, LM, SM, and ES were involved in supervision. VB drafted the manuscript. All authors contributed to revision and approval of the final version of the manuscript.

### Conflict of interest statement

The authors declare that the research was conducted in the absence of any commercial or financial relationships that could be construed as a potential conflict of interest.

## Abbreviations

AIDS: acquired immunodeficiency syndrome
ANC: antenatal care
ART: antiretroviral therapy
CTC: HIV Care and Treatment Centres
DNA: deoxyribonucleic acid
DBS: dried blood spots
EID: Early infant diagnosis
HIV: Human immunodeficiency virus
PSU: primary sampling units
KCMC: Kilimanjaro Christian Medical Centre
NACP: National AIDS Control Program
PCR: polymerase chain reaction
PMTCT: Prevention of mother to child transmission
RCH: Reproductive and child health
SSA: sub- Saharan Africa
TAT: turnaround time
WHO: World Health Organization.
