# Supplementary material for: Accessibility of Early Infant Diagnostic Services by Under-5 Years and HIV Exposed Children in Muheza District, North-East Tanzania
Source: Front Public Health. 2018 May 15;6:139. doi: 10.3389/fpubh.2018.00139 (PMC5962700; doi:10.3389/fpubh.2018.00139)
Supplement: Supplementary file 1 [file Presentation_1.PDF]

Supplementary Material

Accessibility of early infant diagnostic services by under-five years and HIV exposed children in Muheza District, North-East Tanzania

Veneranda M. Bwana[1,2*], Sayoki G. Mfinanga[3], Edgar Simulundu[4], Leonard E.G. Mboera[5] and Charles Michelo[1,6]

[1]University of Zambia, School of Public Health, Lusaka, Zambia
[2]National Institute for Medical Research, Amani Research Centre, Muheza, Tanzania
[3]National Institute for Medical Research, Muhimbili Research Centre, Dar es Salaam, Tanzania
[4]University of Zambia, School of Veterinary Medicine, Department of Disease Control, Lusaka, Zambia
[5]National Institute for Medical Research, Headquarters, Dar es Salaam, Tanzania
[6]Strategic Centre for Health Systems Metrics & Evaluations, University of Zambia, School of Public Health, Lusaka, Zambia

***Correspondence:**
Veneranda Masatu Bwana
Email: vbwana@nimr.or.tz

1. Supplementary data

Materials and Methods

1.1 Study area

The study was conducted in Muheza district in Tanga Region, north-eastern Tanzania (4^0, 45'S; $39^0$00'E). The district covers an area of 1,974 square kilometers with estimated population of about 207,487 (31). About 90% of the people live in the rural areas (35). The district is served by only one district-level general hospital which is called Muheza designated District Hospital (Teule Hospital). The average annual rainfall range from 1000-1500 mm and average temperatures range from 26-32 ^oC. The district has three main climatic belts namely Coastal belt, Lowland plains belt and Mountainous belt. The district has a total of 46 health facilities (one district hospital, 4 health centers and 41 dispensaries); 42 of which provide reproductive and child health services, 36 offer PMTCT services and 28 offer EID services. The prevalence of HIV in the district in 2013 was estimated to be 3.9% (36). However, there were no reported data on HIV prevalence of children below five years. In 2015, the HIV prevalence among pregnant women in Muheza district was 4.0%. The prevalence in other districts ranged from 1.4-3.7% (Lushoto 1.4%, Kilindi 1.7%, Korogwe 3.3%, Handeni 2.1%, Mkinga 2.9% and Pangani 3.9%). The prevalence was 3.7% and 5,5% in Korogwe and Tanga Town councils respectively (37). Muheza district was selected for this study as being among the leading district in Tanga Region with high HIV prevalence among pregnant women (37). The study was conducted in 18 health facilities. The facilities included one district hospital, three health centres (Bulwa, Mkuzi, Ubwari), and 14 dispensaries (Bwembwera, Kibaoni, Kicheba, Kwafungo, Longuza, Magila, Magoda, Misozwe, Mkanyageni, Nkumba, Potwe, Songa,Tongwe and Mtindiro.

1.2 Study design, population and sampling

This was a cross-sectional facility based study that employed a multi-stage sampling approach.

In the first step, we aimed to select 18 out of 46 health facilities in the district. The health facilities defined the primary sampling units (PSUs)/clusters. A list of 46 PSUs by name was obtained from Muheza District Council and numbered from one to 46 according to their geographical location. Then the sampling interval was obtained and 18 health facilities were randomly selected from the list. During the selection process, health facilities that were selected from the list and were found to have no EID services were removed and the next on the list was included. The process was repeated until all 18 facilities that were required for the study were included. In the second step, the study population included all selected mothers/guardians with children below five years born to HIV positive mothers (all HIV exposed children who were not breast feeding for ≥ six weeks) from each PSU. The sample size was calculated based on the formula that accounted for simple random sampling and the design effect which account for between and within cluster variation (38). With the number of clusters available the design effect was adjusted by a factor of 2, at a 95% CI and the desired level of absolute precision was taken at 5%. Based on the recent PMTCT statistics, transmission rates of HIV from mother to child ranges between 20 and 45%. We assumed the highest exposure of infection risk and a response rate of 90% and thus the estimated sample size was 836 children. The study was confined to PMTCT clients; hence the calculated sample size was corrected for this finite population of 836 children. The annual PMTCT clients for Muheza are around 400 women. The correction was done through the following relation.

$$n^* = \frac{n}{1 + \frac{n-1}{N}}$$

Where by: N= finite population, n=is the corrected sample size. Therefore, n*=400/(1+(400-1)/836 = 271. Hence the estimated sample size was 271. Initially the minimum and maximum number of guardian-child pairs in each cluster was set at 20 to 50. But, the proportion to size was set at 5% if the facility has an estimated total number of HIV exposed under fives below 500, and at 20% if it was more than 500 children. The selection was mainly based on availability of EID services at particular health facility. In this study a total of 576 mother/guardian-child pairs were enrolled.
